# Case Report: Non-infectious causes of palmoplantar rashes, what to consider

**DOI:** 10.12688/f1000research.13513.1

**Published:** 2018-01-11

**Authors:** Rashmi Advani, Danit Arad

**Affiliations:** 1 Department of Internal Medicine, Albert Einstein College of Medicine and Montefiore Medical Center, Bronx, NY, 10467, USA

**Keywords:** Palmoplantar skin rash, Medication side-effect, capecitabine

## Abstract

**Background:** Palm and sole skin eruptions have a broad differential diagnosis. It is particularly important to recognize common causes as well as their association with certain chemotherapy regimens such as Capecitabine.

**Case report:** A 79-year-old woman presented with a painful rash on her hands and feet for 1 week. She had metastatic colon cancer and was in her third week of treatment with capecitabine. Her diagnosis was a medication side-effect from chemotherapy. Capecitabine was stopped and she had some clinical improvement over the next two days. She was discharged with oncology follow up for resumption of Capecitabine at a lower dose with improvement in her rash 3 weeks later.

**Discussion:** Skin rashes are a commonly encountered complaint in patients in the inpatient and outpatient setting. It is important to maintain a broad differential diagnosis in those with rashes of the palmoplantar surfaces of the hands and feet. Recognizing skin changes as a possible manifestation of underlying malignancy or a medication side-effect is key in appropriate diagnosis and treatment.

## Introduction

Palmoplantar skin eruption is a commonly encountered diagnosis in the inpatient and outpatient setting. Most likely causes include Type IV hypersensitivity reactions (i.e. contact dermatitis), tinea pedis/manuum, psoriasis, and dyshidrotic dermatitis. These rashes may also be associated with underlying malignancies, especially gastrointestinal malignancies or can be associated with a medication side-effect ^1^. Palmoplantar erythrodysesthesia (PPE), also known as hand-foot syndrome is a toxic, cutaneous side effect of well-associated chemotherapeutic agents, especially capecitabine ^2– 4^. The pathophysiology of this condition is not well understood and is an active area of investigation. It is important to recognize this side effect early in patients treated with oral capecitabine chemotherapy and to differentiate it from similar presentations in other disease entities. We present a case of a woman on chemotherapy for metastatic colon cancer with a palmoplantar rash.

## Case report

A 79- year-old Hispanic woman presented with a one-week history of painful rash on her palms and soles. She reported no recent viral illness, travel, previous rashes, joint pains, new lotion, soap or fabric use. She had metastatic colon cancer previously treated with radiation and hemicolectomy three years prior. She currently completed her second week of Capecitabine therapy (1,250mg/m2 twice a day). She had no other contributory medical history or family history and was on no other medications. She describes never having a similar rash in the past.

**Figure 1.**
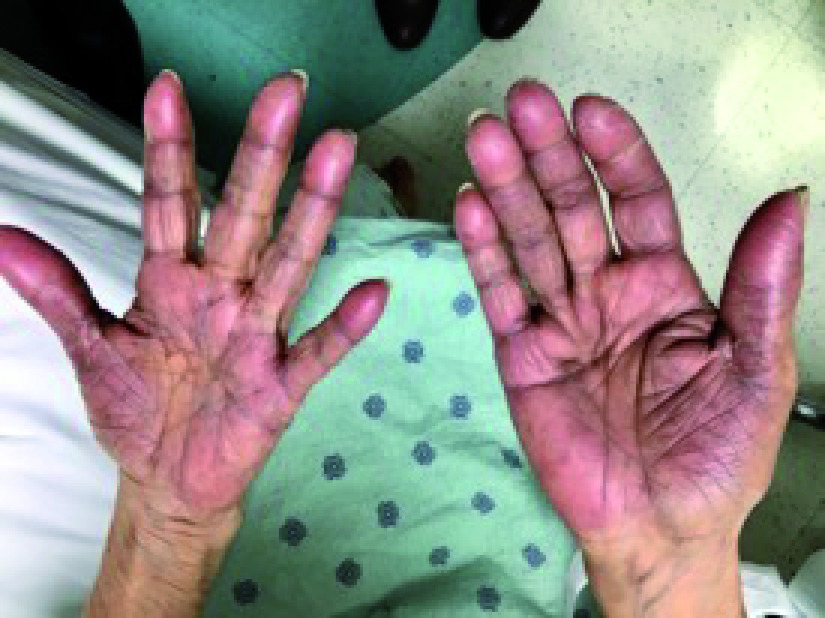
Erythema, swelling, and desquamation of the palmar surfaces.

**Figure 2.**
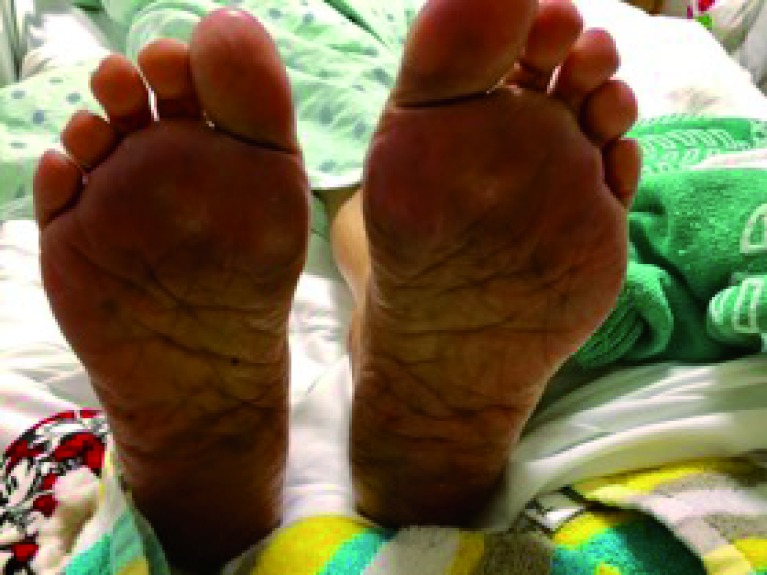
Erythema, swelling, and desquamation of the plantar surfaces.

On physical exam, she was afebrile, normotensive and appeared chronically ill. Her palms and soles were tender to touch, erythematous, and diffusely edematous with desquamation over the fingertips and toes ( Figure 1 and Figure 2). Biochemical testing including complete metabolic panel and complete blood count were normal. Given her recently administered chemotherapy, it was suspected that the patients’ palmoplantar rash was a result of a medication side-effect from Capecitabine. Other less likely diagnoses were contact dermatitis, tinea pedia/mannum, or dyshidrotic dermatitis.

After Capecitabine was stopped, she had mild clinical improvement over the next two days. She was discharged with resumption of Capecitabine at a lower dose (565 mg/m2 twice daily) and had complete clinical resolution of her rash 3 week later.

## Discussion

Palmoplantar skin eruption carries a varied differential diagnosis. Common causes include contact dermatitis, tinea pedis/manuum, psoriasis, dyshidrotic dermatitis and palmoplantar pustulosis. Other palmoplantar rashes such as palmoplantar keratoderma (PPK), Acanthosis Nigricans (AN), Tripe palm, and Acquired Ichthyosis are also associated with underlying malignancies ^5, 6^. PPK presents with a yellow, wax-like hyperkeratosis of the palms and soles. AN, seen in patients with insulin resistance, presents as palmoplantar plaques which can be a sign of internal gastric cancer ^6^. Tripe palm, also associated with gastric and lung malignancies, presents with wrinkled velvety hyperkeratosis of the palmoplantar surfaces ^7^. Lastly, acquired ichthyosis is a symmetric scaling of the skin, associated with Hodgkins lymphoma ^8^.

Since our patient had a temporal relationship between initiation of a new medication and her presentation, it was likely related, if not the cause of her palmoplantar rash. Chemotherapy, such as Capecitabine, is an important cause of palmoplantar skin eruption known as palmoplantar erythrodysesthesia (PPE). It is characterized by pain, swelling and desquamation, which can progress to ulceration and blistering ( Figure 1 and Figure 2). In total, 7% of patients treated with Capecitabine may experience PPE. Other commonly encountered chemotherapy regimens may also cause PPE, such as Cytarabine, Fluorouracil, and Doxorubicin. Treatments include either withdrawal of the chemotherapy or dose reduction, and supportive measures. In our patient’s case, we were limited by not being able to completely stop chemotherapy given her limited therapeutic options; however resuming treatment at a lower dose helped to resolve her symptoms as well as provide a longer life-expectancy.

Common diagnoses aside, medication side-effect and malignancy should be considered in the differential diagnosis of palmoplantar skin eruption to guide appropriate therapy.

## Consent

Written informed consent was obtained from the patient for the publication of the patient’s clinical details and accompanying images.
