# Supplementary material for: Case Report: Non-infectious causes of palmoplantar rashes, what to consider
Source: F1000Res. 2018 Jan 11;7:46. [Version 1] doi: 10.12688/f1000research.13513.1 (PMC5840616; doi:10.12688/f1000research.13513.1)
Supplement: Peer review history

# Peer review: Referee response for version 1

**DOI:** 10.5256/f1000research.14673.r30832

**Published:** 2018-03-05

**Authors:** Regina Fölster-Holst

**Affiliations:** 1 Dermatologische Klinik, Universität Kiel, Kiel, Germany

The authors describe a 79-year old woman presented with palmoplantar rash. This was characterized by painful erythema, edema and desquamation. In this case the history led to the right diagnosis: the woman suffered from metastatic colon cancer and underwent chemotherapy with capecitabine.

 There are other diagnoses to be considered in patients with these clinical findings. Only some of them are mentioned by the authors. For the readers a table with differential diagnoses and typical clinical and anamnestic criteria would have been helpful to differentiate between these diagnoses.

I have read this submission. I believe that I have an appropriate level of expertise to confirm that it is of an acceptable scientific standard.

# Peer review: Referee response for version 1

**DOI:** 10.5256/f1000research.14673.r30831

**Published:** 2018-02-27

**Authors:** Mohamed Badawy Abdel-Naser

**Affiliations:** 1 Department of Dermatology and Venereology, Ain Shams University Hospital, Cairo, Egypt

The case report is well written.

 The title is not addressing the case. Perhaps it can be reformulated to directly refer to the case.

 The statement "however resuming treatment at a lower dose helped to resolve her symptoms as well as provide a longer life-expectancy" - how does reduction of the dose provide longer life-expectancy?

 The provided figures, particularly Figure 2, are not clear.

 Was any local treatment given to the patient?

I have read this submission. I believe that I have an appropriate level of expertise to confirm that it is of an acceptable scientific standard.

# Peer review: Referee response for version 1

**DOI:** 10.5256/f1000research.14673.r30835

**Published:** 2018-02-21

**Authors:** Sidharth Sonthalia

**Affiliations:** 1 Department of Dermatology & Dermatosurgery, Skinnocence: The Skin Clinic & Research Center, Gurgaon, India

1. The title could have been better - "Non-infectious causes of palmoplantar rashes, what to consider" would have been more apt in a case where there was diagnostic confusion based on history and/or examination. In this case, the history of malignancy and the ongoing chemotherapy was forthcoming. Emphasizing upon the fact that one MUST investigate for concurrent malignancy and chemotherapy, to consider non-infectious cause of the hand and foot syndrome (HFS) in a patient who is presenting with the classical clinical features of HFS and history of capecitabine, the most common chemotherapeutic agent responsible for this condition. 
2. Although, despite a strong evidence (history of malignancy and capecitabine) of the etiology of palmo-plantar erythrodysesthesia or HFS, the authors mention that they were considering other less likely diagnoses such as contact dermatitis, dyshidrotic eczema and tinea pedis/manuum. But no confirmatory investigations were done to rule them out. A 10% KOH smear and a punch biopsy should have been taken if these conditions were still being suspected.
3. Ideally, the authors should have mentioned about the state of the nails of the patient also, which often become dystrophic alongwith HFS.
4. It would have been better if the authors had also graded the patient's HFS; in view of the three well-established grading systems in place (WHO, NCI, and one system proposed by Nikolaou et al. ^1^ especially for darker skin types) that have also been documented to guide regarding the therapeutic approach. Similarly, it would have been better if the patient's quality of life (QoL) affected by HFS was quantified using the simple HFS-14 scale. Nikolaou et al have demonstrated that HFS-14 scale may identify differences in QoL impairment between patients with HFS of same clinical grade and may serve as a valuable tool for anticancer treatment management and assessment of clinical efficacy of treatments used for this condition.
5. The authors should have at least made a mention of an important differential of HFS, i.e. HFS reaction (HFSR) that predominantly involves the pressure-prone areas of the soles and typically arises as a reaction to multiple kinase inhibitors (MKI).
6. Since the HFS was not quantified, nor the patient's morbidity due to pain (limitation of instrumental vs self-care activities) has been mentioned, based upon the relatively scarce data on physical examination, the patient seems to qualify for NCI grade 2 HFS. The approach to management in grade 2 has been recommended to stop the chemotherapy for a short duration till the condition improves to grade 1 or 0 (which was done in the current case) followed by resumption at lower doses. However, there are many practical measures and simple drugs/topical applications which if followed could have expedited the improvement such as avoidance of mechanical stress, use of cold compresses, generous moisturization with a urea-containing cream, and potent topical corticosteroids for a short duration. In fact prophylactic oral pyridoxine and celecoxib (COX-2 inhibitor) have also been suggested for a patient with NCI grade 2 HFS. 
7. It is true that in this particular patient, as per the authors' description, uneventful remission occurred within 2-3 weeks of 2-day cessation followed by resumption at low doses of capecitabine, the report would have given out a more wholesome message to the readers if the treatment modalities other than the stoppage of the chemotherapeutic agent were at least mentioned.

I have read this submission. I believe that I have an appropriate level of expertise to confirm that it is of an acceptable scientific standard, however I have significant reservations, as outlined above.

## References

[1] NikolaouVSyrigosKSaifMW: Incidence and implications of chemotherapy related hand-foot syndrome. *Expert Opin Drug Saf*.2016;15(12) : 10.1080/14740338.2016.1238067 1625-1633 10.1080/14740338.2016.1238067 27718746
